# Effects of intrapericardial administration after catheter drainage on malignant pericardial effusion in non‐small cell lung cancer: A real‐world study

**DOI:** 10.1002/cam4.6404

**Published:** 2023-08-02

**Authors:** Jingwen Wei, Zheng Shi, Zhengbo Song

**Affiliations:** ^1^ Department of Clinical Trial The Cancer Hospital of the University of Chinese Academy of Sciences, Zhejiang Cancer Hospital Hangzhou China; ^2^ Postgraduate training base Alliance of Wenzhou Medical University (Zhejiang Cancer Hospital) Hangzhou China

**Keywords:** catheter drainage, intrapericardial administration, malignant pericardial effusion, non‐small cell lung cancer

## Abstract

**Background:**

Malignant pericardial effusion (MPE) is a serious complication of cancer that can be potentially deadly. It usually occurs in advanced or terminal stages of the disease, and as a result, patients with MPE often have a poor prognosis. There is a limited amount of research available that directly compares the effectiveness and safety of intrapericardial drug administration following pericardial drainage versus catheter drainage alone in non‐small cell lung cancer (NSCLC) patients who have MPE.

**Methods:**

We retrospectively included 86 patients with NSCLC with MPE at Zhejiang Cancer Hospital. Survival and recurrence estimates were determined with the Kaplan–Meier method.

**Results:**

We divided the 86 patients with NSCLC into two groups: a pericardial drainage group (34 out of 86, 39.5%) and an intrapericardial administration group (52 out of 86, 60.5%). The response rates were 70.6% and 76.9% (*p* = 0.510), respectively. The median OS was 132.0 and 234.0 days (*p* = 0.579), respectively. The median time to recurrent drainage was 43.0 and 104.0 days (*p* = 0.170), respectively. The incidence of adverse events (AEs) was 44.1% and 61.5% (*p* = 0.113), respectively. The most frequent AEs were pain (27.9%) and fever (24.4%). Additionally, two patients in the intrapericardial administration group died of cardiac arrest.

**Conclusions:**

Compared with catheter drainage alone, intrapericardial medication infusion during catheter drainage did not have significantly different effects. AEs require close monitoring and management.

## 1 INTRODUCTION

With its high incidence rates, lung cancer is responsible for the largest number of cancer‐related deaths across the globe; therefore, it remains a major health concern.^1^ Pericardial effusion in cancer patients can occur through various pathophysiological mechanisms. These include direct extension or metastasis of the underlying malignancy, complications associated with systemic tumor treatment such as radiation therapy or chemotherapy, and opportunistic infections that may arise during antineoplastic therapies.^2^, ^3^ Malignant pericardial effusion (MPE) is a complication of cancer, wherein the accumulation of excessive fluid within the pericardial sac exerts pressure on the heart, thus impeding its ability to function properly. MPE is frequently observed in various types of cancer, affecting 5%–15% of patients, and lung cancer is a major contributor to the development of MPE.^4^, ^5^

Patients diagnosed with non‐small cell lung cancer (NSCLC) and MPE typically have an advanced stage of cancer and a poor outlook for recovery.^6^ Early diagnosis and prompt pericardial drainage can effectively alleviate symptoms. However, pericardiocentesis and catheter drainage have been found to be insufficient in preventing the reoccurrence of MPE.^7^, ^8^ On the basis of pericardial drainage, some patients may undergo intrapericardial drug therapy. Numerous reports of pericardial sclerosis in lung cancer patients with MPE have described treatment with various agents, such as bleomycin (BLM) and carboplatin after drainage.^6^, ^9^ Previous studies have suggested that patient prognosis remains unfavorable despite intrapericardial administration after catheter drainage.^6^, ^9^ Kunitoh et al.^10^ have reported that, although intrapericardial instillation of BLM following pericardial drainage appears to be safe and efficacious in managing MPE, its therapeutic advantage is modest with respect to that of catheter drainage alone. Because patients with MPE generally have poor prognosis, is intrapericardial administration necessary rather than catheter drainage alone? Limited studies have compared the effectiveness and safety of intrapericardial drug administration after pericardial drainage versus catheter drainage alone in NSCLC patients with MPE.

The objective of this study was to compare the safety and efficacy of intrapericardial administration versus catheter drainage alone on MPE in NSCLC, providing valuable clinical insights for future MPE treatment.

## 2 METHODS

### 2.1 Study populations

Between January 2009 and January 2023, we conducted a retrospective analysis of patients with NSCLC who underwent pericardial drainage because of clinical MPE with moderate to high accumulation of fluid at Zhejiang Cancer Hospital. Eligibility criteria for this study included confirmed NSCLC through histological or cytological examination, moderate‐to‐massive MPEs requiring clinical treatment at the time of initial diagnosis, and pericardial effusion cytology indicating malignant cells. Patients with Eastern Cooperative Oncology Group performance status (ECOG PS) scores ranging from 0 to 3 were also eligible, while those with serious comorbidities, active infections, coagulation disorders, or who were pregnant or lactating were excluded. The study protocol was approved by the institutional review board of Zhejiang Cancer Hospital (IRB‐2023‐151) and conducted in accordance with the Declaration of Helsinki (as revised in 2013). As this was a retrospective analysis, individual consent was not required.

### 2.2 Treatment method

Under the guidance of echocardiography, a catheter was inserted into the pericardial space using a percutaneous approach. We divided the patients into a pericardial drainage group and an intrapericardial administration group. Patients in the pericardial drainage group did not receive intrapericardial drug infusion therapy after pericardial drainage, whereas those in the intrapericardial administration group received intrapericardial drug infusion therapy during pericardial drainage. The drugs used for intrapleural therapy included BLM and interleukin 2. In each group, once the initial drainage was completed, the catheter remained in the pericardial space while the drainage tube was removed once the volume of drainage decreased to less than 30 mL per day.

### 2.3 Response and toxicity evaluation

This study employed the MPE response criteria as defined by Paladine et al.^11^ and the United Kingdom Multi‐Centre Study.^12^ Complete response (CR) was defined as no fluid re‐accumulation for at least 30 days post‐treatment, confirmed by clinical examination, chest radiography, or echocardiography. Partial response (PR) was defined as minimal fluid re‐accumulation in the initial 30‐day evaluation period that did not require aspiration. Patients needing re‐aspiration within 30 days were considered treatment failures, while those who died within 30 days after pericardial drainage were marked not evaluated (NE). The control rate was calculated as the sum of CR and PR patients divided by the total enrolled. Overall survival (OS) was measured from the first day of pericardiocentesis to either the patient's death or the last follow‐up date. Time to recurrent drainage (TRD) was defined as the period from the first pericardiocentesis to the next. Adverse events (AEs) were assessed by two or more independent medical professionals using the National Cancer Institute's Common Toxicity Evaluation Criteria and graded 1–5 to analyze their severity.

### 2.4 Statistical analyses

Data analysis was conducted using SPSS (version 25.0; SPSS Inc.) and GraphPad Prism (version 9.2.0; GraphPad). Quantitative variables were compared using the Wilcoxon rank‐sum test, and categorical variables were analyzed using the Pearson chi‐squared or Fisher's exact test. The OS and TRD were evaluated using the Kaplan–Meier method and log‐rank test. Statistical tests were two‐tailed, and a *p* < 0.05 was considered statistically significant. All patients included in the study were followed up, and the follow‐up rate was 100%. The last follow‐up visit was conducted on January 10, 2023.

## 3 RESULTS

### 3.1 Patient characteristics

A total of 86 patients with NSCLC who underwent pericardial drainage were included (Table 1). Among these patients, 34 (39.5%) received only catheter drainage, and 52 (60.5%) received intrapericardial administration. In the overall population, the patients had a median age of 55.52 years (range: 31–78 years) and consisted of 58 (67.4%) men and 28 (32.6%) women. A total of 61 patients (70.9%) had an ECOG PS score of 0–1, and the remaining 25 patients (29.1%) had PS scores in the range of 2–3. A total of 36 patients (41.9%) had a history of smoking; 55 patients (64.0%) were diagnosed with adenocarcinoma; 28 patients (32.6%) were diagnosed with squamous cell carcinoma; 16 patients (18.6%) had a history of surgical treatment; 10 patients (11.6%) had a history of thoracic radiotherapy; and 60 patients (69.8%) had a history of chemotherapy. The baseline characteristics were comparable between the pericardial drainage group and the intrapericardial administration group, and no statistically significant difference was observed.

**TABLE 1:** Baseline characteristics of 86 patients with NSCLC with MPE.

Characteristic	Patients, *n* (%)	Pericardial drainage group, *n* (%)	Intrapericardial administration group, *n* (%)	*p*‐value
Median age at drainage (years)	55.52 (31–78)	55.74 (31–72)	55.38 (32–78)	0.811
Sex				
Male	58 (67.4)	21 (61.8)	37 (71.2)	0.364
Female	28(32.6)	13 (38.2)	15 (28.8)	
ECOG PS				
0 or 1	61 (70.9)	22 (64.7)	39 (75.0)	0.304
2 or 3	25 (29.1)	12 (35.3)	13 (25.0)	
Smoking history				
Yes	36 (41.9)	14 (41.2)	22 (42.3)	0.917
No	50 (58.1)	20 (58.8)	30 (57.7)	
Histologic type				
Adenocarcinoma	55 (64.0)	21 (61.8)	34 (65.4)	0.165
Squamous cell carcinoma	28 (32.6)	13 (38.2)	15 (28.8)	
Other	3 (3.5)	0 (0)	3 (5.8)	
Bone metastasis				
Yes	69 (80.2)	28 (82.4)	41 (78.8)	0.690
No	17 (19.8)	6 (17.6)	11 (21.2)	
Brain metastasis				
No	81 (94.2)	33 (97.1)	48 (92.3)	0.653
Yes	5 (5.8)	1 (2.9)	4 (7.7)	
Liver metastasis				
No	83 (96.5)	32 (94.1)	51 (98.1)	0.706
Yes	3 (3.5)	2 (5.9)	1 (1.9)	
Previous surgery				
No	70 (81.4)	28 (82.4)	42 (80.8)	0.854
Yes	16 (18.6)	6 (17.6)	10 (19.2)	
Previous thoracic radiotherapy				
No	76 (88.4)	30 (88.2)	46 (88.5)	>0.999
Yes	10 (11.6)	4 (11.8)	6 (11.5)	
Previous chemotherapy				
No	26 (30.2)	13 (38.2)	13 (25.0)	0.191
Yes	60 (69.8)	21 (61.8)	39 (75.0)	

Abbreviations: ECOG PS, Eastern Cooperative Oncology Group performance status; MPE, malignant pericardial effusion; NSCLC, non‐small cell lung cancer.

### 3.2 Treatment efficacy and patient survival

Effusion volumes ranged from 50 to 4460 mL (median: 907.5 mL), and pericardial drainage durations ranged from 1 to 34 days (median: 8.2 days) (Table 2). Among all 86 patients, the response rate was 74.4%. Of the patients in the pericardial drainage group, 2 (5.9%) achieved CR, 22 (64.7%) achieved PR, 5 (14.7%) experienced treatment failure, and 5 (14.7%) were NE, thus resulting in a response rate of 70.6% (24 out of 34). Of the patients in the intrapericardial administration group, 12 (23.1%) achieved CR, 28 (53.8%) achieved PR, 4 (7.7%) experienced treatment failure, and 8 (15.4%) were NE during follow‐up, thus resulting in a response rate of 76.9% (40 out of 52). There was no significant difference observed in the response rate (*p* = 0.510).

**TABLE 2:** Treatment profile and evaluation of responses.

Characteristic	Patients, *n* (%)	Pericardial drainage group, *n* (%)	Intrapericardial instillation group, *n* (%)
Median drainage volume (mL)	907.5 (50–4460)	799.9 (140–4460)	977.9 (50–2200)
Duration of drainage (days)	8.2 (1–34)	7.8 (1–21)	8.5 (2–34)
Treatment response			
Not evaluated	13 (15.1)	5 (14.7)	8 (15.4)
Evaluated			
Complete response	14 (16.3)	2 (5.9)	12 (23.1)
Partial response	50 (58.1)	22 (64.7)	28 (53.8)
Treatment failure	9 (10.5)	5 (14.7)	4 (7.7)

**FIGURE 1:**
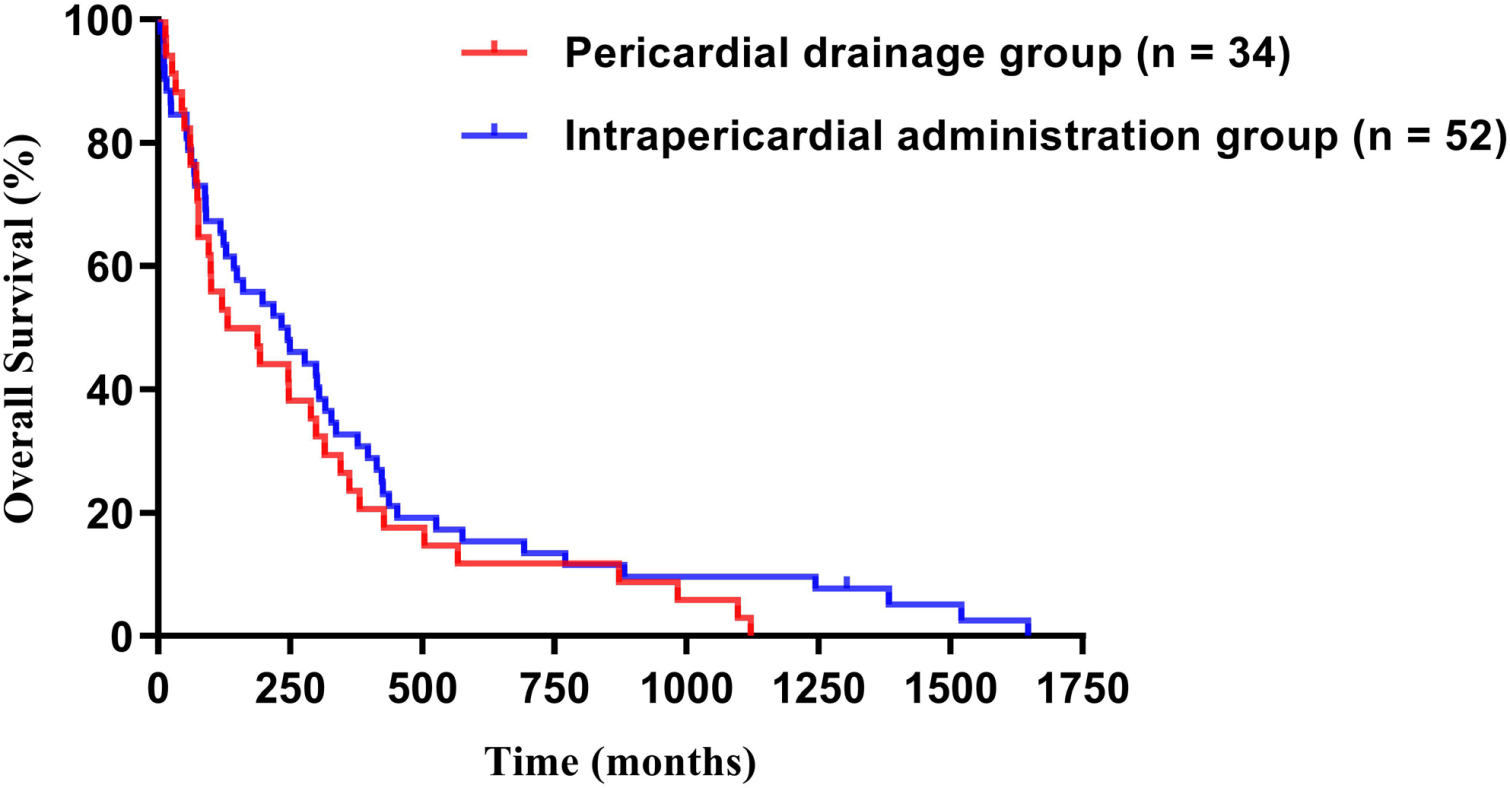
OS of patients with NSCLC in the pericardial drainage group (*n* = 34) and intrapericardial administration group (*n* = 52). The OS did not differ between groups (OS, 132.0 vs. 234.0 days, *p* = 0.305). NSCLC, non‐small cell lung cancer; OS, overall survival.

**FIGURE 2:**
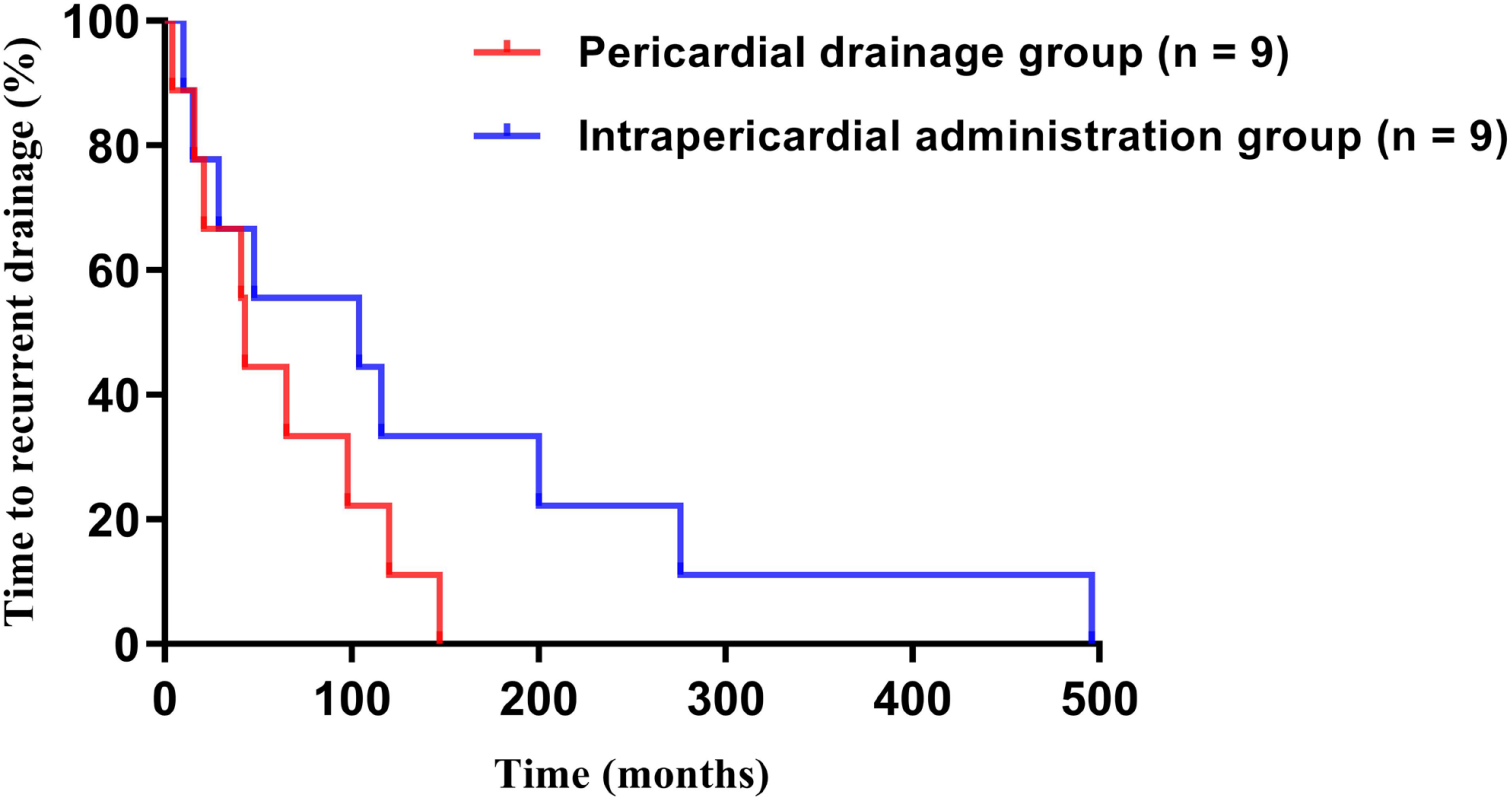
TRD of patients with NSCLC in the pericardial drainage group (*n* = 9) and intrapericardial administration group (*n* = 9). TRD did not differ between groups (TRD, 43.0 vs. 104.0 days, *p* = 0.170). NSCLC, non‐small cell lung cancer; TRD, time to recurrent drainage.

In all 86 patients, the median OS was 198.0 days (103.5–292.5 days). The median OS in the pericardial drainage group was 132.0 days (0.6–263.4 days), and that in the intrapericardial administration group was 234.0 days (96.2–371.8 days). There was no significant difference in OS between groups (*p* = 0.305, Figure 1). A total of 18 patients (18 out of 86, 20.9%) required repeat drainage after the first drainage: 9 patients (9 out of 34, 26.5%) in the pericardial drainage group and 9 (9 out of 52, 17.3%) in the intrapericardial administration group. Among these 18 patients, the median TRD was 48.0 days (2.3–93.7 days). The median TRD in the pericardial drainage group was 43.0 days (37.2–48.8 days), and that in the intrapericardial administration group was 104.0 days (0.0–267.6 days). There was no significant difference in TRD between groups (*p* = 0.170, Figure 2).

**FIGURE 3:**
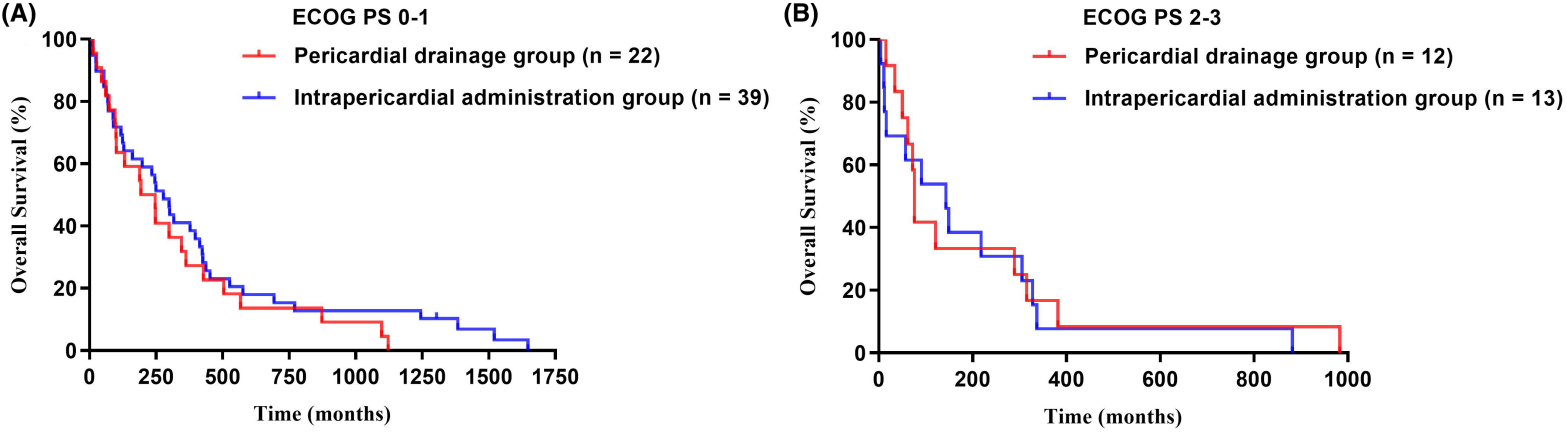
(A) OS of patients with a PS score of 0–1 in the pericardial drainage group (*n* = 22) and intrapericardial administration group (*n* = 39). The OS did not differ between groups (OS, 193.0 vs. 278.0 days, *p* = 0.351). (B) OS of patients with a PS score of 2–3 in the pericardial drainage group (*n* = 12) and intrapericardial administration group (*n* = 13). The OS did not differ between groups (OS, 76.0 vs. 143.0 days, *p* = 0. 800). ECOG PS, Eastern Cooperative Oncology Group performance status; NSCLC, non‐small cell lung cancer; OS, overall survival.

In patients with a PS score of 0–1, 22 patients received pericardial drainage alone, while 39 patients received intrapericardial administration of medication after drainage. The median OS in the pericardial drainage group was 193.0 days (60.8–325.2 days), and that in the intrapericardial administration group was 278.0 days (196.0–360.0 days). There was no significant difference in OS between groups (*p* = 0.351, Figure 3A). In patients with a PS score of 2–3, 12 patients received pericardial drainage alone, while 13 patients received intrapericardial administration of medication after drainage. The median OS in the pericardial drainage group was 76.0 days (69.3–82.7 days), and that in the intrapericardial administration group was 143.0 days (35.0–251.0 days). There was no significant difference in OS between groups (*p* = 0.800, Figure 3B).

### 3.3 Adverse events

Table 3 outlines the AEs observed in both the pericardial drainage group and the intrapericardial administration group. The overall incidence of AEs was 54.7% (47 out of 86). The percentage of AEs in the pericardial drainage group was 44.1% (15 out of 34), and that in the intrapericardial administration group was 61.5% (32 out of 52) (*p* = 0.113). The most common AEs were pain (24 out of 86, 27.9%) and fever (21 out of 86, 24.4%). The cardiac‐associated AEs observed were sinus tachycardia, ventricular tachycardia, ventricular arrhythmia, atrial fibrillation, and palpitations. Notably, two patients in the intrapericardial administration group died of cardiac arrest. Eleven (12.8%) patients experienced cardiac AEs: two (2 out of 34, 5.8%) in the pericardial drainage group and nine (9 out of 52, 17.3%) in the intrapericardial instillation group. The difference in the occurrence of AEs observed between groups was not statistically significant.

**TABLE 3:** Frequency of treatment‐associated adverse events.

Adverse events	Pericardial drainage group			Intrapericardial instillation group			*p*‐value
	Grade 1	Grade 2	Grade 3	Grade 1	Grade 2	Grade 3	
Pain	5	4	0	13	2	0	0.234
Fever	6	0	0	11	3	1	0.469
Sinus tachycardia	0	0	0	2	0	0	0.561
Ventricular tachycardia	0	1	0	1	1	0	>0.999
Ventricular arrhythmia	0	0	0	0	1	0	>0.999
Atrial fibrillation	0	2	0	0	1	0	0.706
Palpitations	0	0	0	2	0	0	0.561
Malaise	0	1	0	2	0	0	0.300
Nausea	0	1	0	2	0	0	0.300
Edema face	1	1	0	0	0	0	0.153

*Note*: The data are presented as the number of patients. Two patients in the intrapericardial administration group died of cardiac arrest.

## 4 DISCUSSION

Pericardiocentesis with drainage and intrapericardial chemotherapy has been widely used in the treatment of moderate to large MPE. However, relatively little research has assessed isolated pericardial drainage and intrapericardial drug administration in NSCLC. This study examined the management of MPE caused by NSCLC and compared the effectiveness and safety of catheter drainage alone versus intrapericardial drug administration for treating MPE in NSCLC patients.

MPE is a severe complication of malignancy that often occurs in advanced or terminal stages of the disease, indicating a poor prognosis for patients with symptomatic pericardial effusion. Catheter drainage has been reported to be effective in controlling benign pericardial effusion, according to Kopecky et al.^13^ Apodaca‐Cruz et al.^8^ suggested that catheter drainage could be considered as an initial treatment option, especially for patients with a poor prognosis, and reported a median OS of 40.3 ± 7.9 weeks after pericardiocentesis. Studies have also demonstrated the use of intrapericardial instillation for delivering drugs such as BLM,^14^, ^15^ carboplatin,^9^, ^16^, ^17^ cisplatin,^18^ and mitomycin C^19^ to patients with MPE. One study has reported that intrapericardial instillation of carboplatin effectively controlled MPE in nine of 10 patients with NSCLC.^16^ In a study involving 21 patients with symptomatic MPE associated with lung cancer, intrapericardial administration of carboplatin resulted in a 66.7% control rate of MPE 30 days after treatment and a median survival time of 71 days.^9^ BLM instillation was performed in 31 cancer patients with cardiac tamponade, resulting in non‐fatal complications in three patients and one patient experiencing relapse. The OS rate was less than 10%, and the median survival time was 104 days.^20^ A Phase II trial involving 22 NSCLC patients found that local BLM instillation after complete pericardial drainage achieved a 95% control rate of pericardial effusion and a median survival time of 17.9 weeks.^6^ The JCOG9811 study has compared pericardial drainage alone to drainage followed by intrapericardial instillation of BLM, and observed median survival times of 79 and 119 days, respectively; although intrapericardial BLM appeared to be effective, its therapeutic advantage was modest.^10^ The above studies have indicated that patients with MPE have poor prognosis even with treatment. In this study in 86 patients, the response rate was 74.4%, a value comparable to those in previous studies in NSCLC patients. In the pericardial drainage group and intrapericardial administration group, the response rate was 70.6% and 76.9% (*p* = 0.510), respectively. The median OS was 132.0 and 234.0 days, respectively. The median TRD was 43.0 and 104.0 days, respectively. However, no significant difference was found between groups in OS and TRD. Furthermore, considering that patients with different PS scores may have different prognoses, separate analyses were performed for patients with PS scores of 0–1 and 2–3. The results still showed that receiving pericardial drainage alone or intrapericardial administration of medication after drainage had no statistically significant impact on the OS of patients. The therapeutic potential of drainage alone for this severe oncological problem may be limited. Although pericardial instillation therapy combined with drainage may appear to be more effective than drainage alone, its therapeutic benefits appear to be limited.

Previous studies have shown that intrapericardial administration therapy infrequently results in AEs of Grade 2 or higher, and the most commonly reported AEs are pain and fever.^6^, ^9^, ^17^ The incidence of AEs in the pericardial drainage group and intrapericardial administration group was 44.1% (15 out of 34) and 61.5% (32 out of 52), respectively (*p* = 0.113). The most frequent AEs were pain and fever. Two patients in the intrapericardial instillation group died of cardiac arrest. Cardiac events are of particular concern during the treatment of MPE, particularly in patients undergoing intrapericardial instillation therapy.

The current analysis had several limitations. First, as this study was retrospective in nature, we were limited in our ability to investigate late cardiac complications, which could have potentially introduced bias into our results. Second, the study compared intrapericardial administration with pericardial drainage alone but did not specifically restrict the intrapericardial drugs used, thus potentially leading to bias. Future research may explore the efficacy of different drugs used for intrapericardial treatment. Third, a potential limitation of our exploratory study is that it included patients with a wide range of clinical conditions and prognoses, including those with cancerous pericarditis; consequently, the findings reflect the outcomes of real world. Fourth, although the drainage method remained consistent, the utilization of systemic anticancer agents and healthcare approaches varied over time, and their application might have impacted the survival before and after the treatment for MPE.

## 5 CONCLUSIONS

Compared with catheter drainage alone, intrapericardial medication infusion during catheter drainage was not found to have significantly different effects or safety. AEs required careful monitoring and appropriate management. A future prospective study is needed to assess the safety and efficacy of pericardial drainage and subsequent intrapericardial perfusion therapy.

## AUTHOR CONTRIBUTIONS

**Jingwen Wei:** Data curation (lead); investigation (equal); validation (lead); writing – original draft (lead). **Zheng Shi:** Data curation (supporting); investigation (equal). **Zhengbo Song:** Conceptualization (lead); funding acquisition (lead); writing – review and editing (lead).

## FUNDING INFORMATION

The study was supported by the Medical Scientific Research Foundation of Zhejiang Province (grant number: 2022KY653), and Zhengbo Song was sponsored by the Zhejiang Provincial Program for the Cultivation of High‐level Innovative Health Talents.

## CONFLICT OF INTEREST STATEMENT

The authors have no relevant financial or non‐financial interests to disclose.

## ETHICS STATEMENT

The study was approved by the Institutional Ethics Committee at Zhejiang Cancer Hospital (IRB‐ 2023‐151) and individual consent for this retrospective analysis was waived.

## Data Availability

The data that support the findings of this study are available from the corresponding author upon reasonable request.
